# Environmental Transport and Transformation of Pollutants

**DOI:** 10.3390/toxics13121028

**Published:** 2025-11-27

**Authors:** Xiaoxia Lu

**Affiliations:** Ministry of Education Laboratory for Earth Surface Processes, College of Urban and Environmental Sciences, Peking University, Beijing 100871, China; luxx@urban.pku.edu.cn

## 1. Introduction

In recent years, study on pollutant environmental transport and transformation has witnessed remarkable progress driven by interdisciplinary integration and technological innovation. Research focus has expanded from conventional pollutants to emerging contaminants [1]. High-resolution mass spectrometry and genomic technologies have enabled the accurate identification and quantification of trace emerging pollutants and microbes, facilitating the shift from “detection” to “mechanism exploration” [2,3]. The recognition of multi-media transport processes has deepened, with studies revealing the complex exchange of pollutants among the atmosphere, water, soil, and biota [4]. Numerical modeling techniques have advanced significantly, including the development of coupled reactive transport models for complex pollution systems and the integration of machine learning to improve prediction accuracy [5,6].

Despite these advances, critical knowledge gaps remain. For instance, the synergistic transport and transformation mechanisms of coexisting pollutants are not fully understood, particularly the coupling effects of chemical, physical, and biological processes in complex environmental matrices. The environmental behaviors of emerging pollutants are not well characterized, including their transformation pathways across different media and the ecological risks of transformation products. Existing numerical models often lack integration of multi-scale processes and real-time data assimilation, leading to uncertainties in risk assessment.

## 2. An Overview of Published Articles

This Special Issue comprises 12 studies (11 research articles and 1 review) covering core themes of pollutant adsorption, microbial degradation, nanoparticle fate, multi-phase partitioning, risk assessment, and toxicity. These works target and fill several key knowledge gaps identified in the field, as summarized below.

Zhang et al. (2024) (contribution 1) investigated pyrene (a PAH) and arsenite (As(III)) adsorption by micro/nano carbon black (C 94.03%, spherical, 100–200 nm) and iron oxide (hematite, irregular rod-shaped, ~1 μm long). The key findings were as follows: (1) carbon black preferentially adsorbed pyrene (24 h adsorption capacity: 283.23 μg/g; pseudo-second-order rate constant: 0.016 mg/(g·h)), while iron oxide adsorbed As(III) (24 h adsorption capacity: 3.45 mg/g; rate constant: 0.814 mg/(g·h)), with chemical reactions as the main mechanism; and (2) As(III) reduced pyrene adsorption on carbon black (effect strengthened with increasing As(III) concentration), while pyrene enhanced As(III) adsorption on iron oxide. This fills the gap in co-pollutant interaction during adsorption, guiding combined PAH-As remediation.

Liu et al. (2024) (contribution 2) explored benzene and toluene biodegradation under sulfate-reducing conditions using groundwater samples from contaminated sites. By the end of the study (day 90), about 99% benzene and 96% toluene were removed. During the study, bacterial community richness initially decreased but subsequently increased over time. Key degraders of benzene and toluene were identified as *Pseudomonas*, *Janthinobacterium*, *Novosphingobium*, *Staphylococcus*, and *Bradyrhizobium*. This fills the gap in microbial community dynamics during sulfate-driven BTEX biodegradation, providing a basis for biostimulation strategies.

Khan et al. (2024) (contribution 3) analyzed ZnO-NP aggregation in simulated water with perfluorooctanoic acid (PFOA), humic acid (HA), and electrolytes (NaCl, CaCl_2_) over 3 weeks. ZnO-NP size increased from 162.4 nm (1 day) to >10 μm (3 weeks) and Zeta potential decreased from −47.2 mV (1 day) to −0.2 mV (3 weeks). HA and PFOA dispersed ZnO-NPs via aliphatic carbon interactions and complex structures, while electrolytes altered surface charge. This clarifies how multiple coexisting substances regulate ZnO-NP aggregation, addressing the gap in nanoparticle fate prediction.

Khan et al. (2025) (contribution 6) explored how the presence of coexisting organic pollutants (like tetrabromobisphenol-A (TBBPA)), electrolytes (NaCl and CaCl_2_), humic acid (HA), and bovine serum albumin (BSA) in water affected the behavior of ZnO-NPs. They found that TBBPA and salts promoted aggregation via cation bridging and hydrophobic interactions, while HA/BSA enhanced dispersion by modifying zeta potential. This further expands understanding of ZnO-NP behavior in complex systems.

Yang et al. (2024) (contribution 4) studied estrogen (estrone E1, 17β-estradiol E2, estriol E3) partitioning in Taiwan’s Wulo Creek (impacted by feedlot wastewater), separating samples into suspended particulate matter (SPM), colloidal, and soluble phases. The results showed that estrogens dominated in the soluble phase (85.8–87.3%), followed by colloids (12.7–14.2%); Log K_COC_ (4.72–4.77 L/kg-C) was much higher than Log K_OC_/Log K_POC_ (2.02–3.40 L/kg-C), indicating that colloids play a critical role in estrogen transport. This addresses the gap of ignoring colloidal phases, improving ecological risk assessment accuracy.

Hou et al. (2024) (contribution 5) synthesized a covalent organic-framework-modified biochar (RH-COF) for cadmium (Cd^2+^) adsorption. The modified material showed a 14-fold increase in Cd^2+^ capacity (from 4.20 to 58.62 mg/g) due to elevated nitrogen (from 0.96% to 5.40%) and oxygen (from 15.50% to 24.08%) content. Adsorption followed pseudo-second-order kinetics and Langmuir isotherms, with mechanisms including surface complexation, chelation, and electrostatic adsorption. This addresses the gap in low-efficiency Cd remediation by developing a high-performance adsorbent.

Kawichai et al. (2025) (contribution 7) developed a machine learning (ML) model to predict long-term PM_2.5_ concentrations in upper northern Thailand (impacted by biomass burning). The best ML prediction model was selected considering root mean square error (RMSE), mean prediction error (MPE), relative prediction error (RPE), and coefficient of determination (R^2^). Using 2011–2020 data (PM_10_, CO_2_, O_3_, fire hotspots, air pressure, rainfall, relative humidity, temperature, wind direction, and wind speed), the random forest (RF) model outperformed others (RMSE: 6.82 μg/m^3^, R^2^: 0.93). This fills the gap in long-term PM_2.5_ retrospective data, supporting health effect studies.

Cai et al. (2025) (contribution 9) measured 10 organophosphate esters (OPEs) in 46 homes, 12 offices, 6 dormitories, and 60 private cars in Guangzhou. Among the four microenvironments, private vehicles exhibited the highest total OPE concentrations (ΣOPEs), with an average of 264.89 ng/m^3^—statistically significantly higher than the other three environments (*p* < 0.05). In homes, offices, and student dormitories, tris(2-chloroethyl) phosphate (TCEP) and tris(2-chloropropyl) phosphate (TCPP) dominated the OPE mixture, accounting for 56% and 34% of ΣOPEs, respectively. By contrast, private cars were characterized by elevated levels of TCPP (68% of ΣOPEs) and tris(1,3-dichloro-2-propyl) phosphate (TDCP, 12%). This addresses the gap in OPE data for car microenvironments.

Shi et al. (2025) (contribution 10) investigated the adsorption, diffusion, and advection–dispersion behavior of ^99^Tc in the following three types of rocks: granite, clay rock, and mudstone shale. They found that the three types of rocks had no significant adsorption effect on ^99^Tc. The anion exclusion during diffusion and advection–dispersion processes could block small “channels”, causing some nuclide migration to lag, but accelerated the nuclide migration rate in larger “channels”. In addition, parameters characterizing the size of anion exclusion in different migration behaviors, such as effective diffusion coefficient (De) and immobile liquid region porosity (θim), were fitted and obtained, guiding radioactive waste disposal.

Berrios-Rolon et al. (2025) (contribution 11) studied three low-molecular-weight polycyclic aromatic hydrocarbons (PAHs), naphthalene (NAP), phenanthrene (PHEN), and anthracene (ANT), in Puerto Rico’s Cucharillas Marsh. ∑3PAH concentrations ranged from 7.4 to 2198.8 ng/L, with higher wet-season levels (mean = 745.79 ng/L) than dry-season levels (mean = 186.71 ng/L). A predominant pyrogenic origin was identified, with robust PHEN–ANT correlation (r = 0.824) confirming shared combustion-related sources. Acute ecological risk was low (HQ < 0.01), but chronic risks from PHEN/ANT were noted. This fills the gap in PAH data for tropical urban wetlands.

Berrios-Rolon et al. (2025) (contribution 8) provided a systematic review offering new perspectives on the distribution, sources, and ecotoxicological impacts of PAHs in freshwater systems. They investigated spatiotemporal variability across geographic regions, examining both anthropogenic and natural sources, as well as the mechanisms driving PAH transport and fate. Special attention was given to the ecotoxicological effects of PAHs on freshwater organisms, including bioaccumulation, endocrine disruption, and genotoxicity. They identified knowledge gaps and proposed an interdisciplinary risk assessment framework, serving as a roadmap for future freshwater PAH research.

Xu et al. (2025) (contribution 12) assessed the cytotoxic effects of three typical organic iodides (1,2-diiodoethane, 1,3-diiodopropane, and 1,4-diiodobutane) identified in shale gas extraction wastewater on human hepatocellular carcinoma (HepG2) cells. All three diiodoalkanes exhibited significant toxic effects on HepG2 cells at a concentration of 25 μM. They induced abnormal expression of genes associated with the extracellular space, extracellular matrix (ECM), and endoplasmic reticulum (ER) in HepG2 cells, and triggered excessive intracellular ROS production. This fills the gap in diiodoalkane toxic mechanisms.

## 3. Conclusions

This Special Issue showcases the diversity and depth of current research on pollutant environmental transport and transformation, addressing critical knowledge gaps through empirical and theoretical contributions. The studies not only advance scientific understanding but also provide practical tools for pollution remediation and risk management. As the field evolves, interdisciplinary collaboration and technology-driven innovation will remain key to tackling emerging environmental challenges and safeguarding ecological and human health.

## Data Availability

Not applicable.
